# Dysregulation of complement and coagulation pathways: emerging mechanisms in the development of psychosis

**DOI:** 10.1038/s41380-021-01197-9

**Published:** 2021-07-05

**Authors:** Meike Heurich, Melanie Föcking, David Mongan, Gerard Cagney, David R. Cotter

**Affiliations:** 1 School of Pharmacy and Pharmaceutical Sciences, Cardiff University, Cardiff, UK; 2 Department of Psychiatry, Royal College of Surgeons in Ireland, Dublin, Ireland; 3 School of Biomolecular and Biomedical Science, Conway Institute, University College Dublin, Dublin, Ireland

**Keywords:** Neuroscience, Biological techniques

## Abstract

Early identification and treatment significantly improve clinical outcomes of psychotic disorders. Recent studies identified protein components of the complement and coagulation systems as key pathways implicated in psychosis. These specific protein alterations are integral to the inflammatory response and can begin years before the onset of clinical symptoms of psychotic disorder. Critically, they have recently been shown to predict the transition from clinical high risk to first-episode psychosis, enabling stratification of individuals who are most likely to transition to psychotic disorder from those who are not. This reinforces the concept that the psychosis spectrum is likely a central nervous system manifestation of systemic changes and highlights the need to investigate plasma proteins as diagnostic or prognostic biomarkers and pathophysiological mediators. In this review, we integrate evidence of alterations in proteins belonging to the complement and coagulation protein systems, including the coagulation, anticoagulation, and fibrinolytic pathways and their dysregulation in psychosis, into a consolidated mechanism that could be integral to the progression and manifestation of psychosis. We consolidate the findings of altered blood proteins relevant for progression to psychotic disorders, using data from longitudinal studies of the general population in addition to clinical high-risk (CHR) individuals transitioning to psychotic disorder. These are compared to markers identified from first-episode psychosis and schizophrenia as well as other psychosis spectrum disorders. We propose the novel hypothesis that altered complement and coagulation plasma levels enhance their pathways’ activating capacities, while low levels observed in key regulatory components contribute to excessive activation observed in patients. This hypothesis will require future testing through a range of experimental paradigms, and if upheld, complement and coagulation pathways or specific proteins could be useful diagnostic or prognostic tools and targets for early intervention and preventive strategies.

## Introduction

Psychotic disorders such as schizophrenia are among the most severe mental disorders, with large individual and societal costs [1]. Early identification and intervention are associated with improved symptomatic and functional outcomes [2, 3]. The liability to psychosis likely exists on a spectrum within the general population [4, 5].

Operationalized criteria for the clinical high-risk (CHR) state [6] serve to identify vulnerable groups at enhanced risk of psychotic disorders [3]. Identification of risk factors for development of psychosis in high-risk individuals is a key aspect of active research [7, 8]. Prognostication of risk is difficult based on clinical symptoms alone, with only ~20–35% of CHR individuals developing psychosis at 3 years. There is thus an urgent need to identify clinically translatable early biomarkers of psychosis risk [9]. Recently, baseline plasma proteomic biomarkers, predominantly components of the complement and coagulation pathways, have been found to accurately discriminate between CHR individuals who do and do not go on to develop a first psychotic episode [10]. This suggests that dysregulation of these systems could play a crucial role in early detection and may provide insights into the early pathophysiology of psychosis.

Here, we propose that the complement and coagulation systems have a central role in the development of psychosis phenotypes. We review existing evidence for immune and coagulation dysfunction in the blood associated with the psychosis phenotypic spectrum. We then focus on the physiological roles of complement and coagulation before examining how dysfunction of these pathways may give rise to pathology. We integrate novel findings into a refinement of the prevailing “two-hit” hypothesis, where early genetic and/or environmental developmental disruptions to the developing central nervous system (CNS) (“first-hit”) increase the vulnerability of the individual to subsequent, late environmental disruptions (“second-hit”), leading to the development of CNS manifestation [11–13]. This proposed theory integrates complement and coagulation dysregulation that can lead to immune activation, contributing to the development of psychotic disorder. Finally, we consider the potential implications in relation to treatment of psychotic disorders and propose directions for future research.

## Evidence of complement and coagulation dysfunction in psychosis

The pathophysiology of psychosis is complex, with multiple and heterogeneous biological and environmental factors identified across the developmental lifespan [14]. Inflammation and immune activation are implicated in psychotic disorders such as schizophrenia and bipolar disorder and other major mental disorders. A strong genetic association was established by genome-wide association studies between the major histocompatibility complex (MHC) and schizophrenia [15], including the MHC genetic locus encoding some complement components. This association was partly explained by allelic structural variation of the complement component 4 (C4) gene [16]. In the same study, C4 RNA expression was found to be increased in postmortem brain samples from patients with schizophrenia compared to controls and on immunohistochemical analysis was noted to localize to neurons and synapses [16]. Overexpression of C4A in mice revealed reduced cortical synapse density, increased microglial engulfment of synapses, as well as altered mouse behavior [17]. Transcriptomic studies identified cross-tissue gene signatures in both brain and blood associated with schizophrenia, including pathways mediating immune functions, for instance, differentially expressed complement receptors and regulators [18]. Blood plasma biomarker studies reported elevated complement activation in first-episode psychosis (FEP) [19], with inconsistent results regarding total C3 and unaltered C4 plasma levels [20, 21], although C4B variant specific as well as C4B and C4-S variant deficiency have been observed [22]. Several studies analyzing complement pathway-specific activity have indicated increased complement activation in schizophrenia patients, although inconsistent findings have also been reported [20, 23]. Other links between complement and coagulation pathway dysregulation and schizophrenia include enhanced coagulation activation [24, 25], altered blood protein levels, and abnormal protein phosphorylation [26] and changes in the fibrinolytic [27] and anticoagulant [28] pathways.

## The physiological role of complement and coagulation pathways

The complement and coagulation pathways (KEGG: hsa04610) are central to host defense against pathogen infection and injury. The complement system is a key component of the innate immune defense. It is composed of plasma and cell-bound proteins, which are activated via three distinct pathways: the classical pathway (CP, antibody–antigen complex), lectin pathway (LP, carbohydrates), and alternative pathway (AP, contact activation), which converge at the level of C3 activation leading to formation of a membrane-attack complex [29, 30]. Innate immune defense mechanisms can trigger coagulation to limit the invasiveness of the pathogens as well as respond to injury [31], through fibrin formation either by intrinsic (contact activation) or extrinsic (tissue factor (TF)) pathway activation [32]. This is subsequently regulated by plasminogen activation-mediated fibrinolysis [33]. Increased evidence of molecular crosstalk between coagulation and inflammation [34] suggests that coagulation activation can increase inflammation, which in turn amplifies coagulation [35].

## Complement and coagulation protein synthesis

In response to infection or injury, immune cells express inflammatory mediators such as cytokines, causing hepatocytes to secrete acute phase proteins, including the complement and coagulation proteins [36, 37]. The acute phase response to cytokine signaling induces profound changes in the plasma proteome [38]. There is evidence of increased levels of IL-6 in schizophrenia patients [39], and other proinflammatory cytokines were observed to be increased in CHR individuals who later developed psychosis compared to those who did not [40], linking inflammatory cytokines to complement and coagulation protein expression. While the liver is the primary site of synthesis for acute phase proteins, extrahepatic synthesis of complement and coagulation components has been reported at serum‐restricted sites [41].

## Complement and coagulation pathway activation

Complement and coagulation proteins commonly circulate in plasma as zymogens or procofactors that must be converted into active enzymes or cofactors via limited proteolysis through pathway-specific activation mechanisms [42]. Complement activation relies on recognition of localized molecular patterns, which triggers proteolysis, while regulatory components support clearance of inflammatory mediators after elimination of the trigger [43]. Once triggered, a powerful cascade-like activation of proteins result in: opsonization (C3b, iC3b (AP); C4b (CP, LP)) of pathogens or cells marked for phagocytic clearance; generation of signaling molecules promoting chemotaxis of leukocytes (C5a); and direct cell lysis and killing (C5b-9) [30, 44]. In addition to pathogen killing, complement activation also promotes clearance of immune complexes, or apoptotic cells, and acts as an interface between innate and adaptive immunity [45, 46]. The coagulation TF pathway requires exposure of TF with plasma [47], while the intrinsic (FXII-mediated) pathway is triggered by contact with anionic surfaces, both converging at thrombin-mediated fibrin clot formation [48]. Coagulation is linked to innate immunity as it also contributes to host–pathogen defenses [32] by limiting pathogen dissemination and supporting pathogen killing [31]. Dysregulation of the complement and coagulation systems is caused by continuous activation of either proteolytic pathway (e.g., through unresolved, prolonged infection) or inadequate regulation (e.g., as a result of inherited or acquired deficiencies). This may lead directly to pathology [44] or contribute to infection-related complications such as thrombosis [31]. Pathway-specific dysregulation leads to different phenotypes seen in several diseases, which further vary with genetic or nongenetic risk factors predisposing to disease [49, 50].

## Molecular crosstalk of complement and coagulation proteins

Expression level differences in complement and coagulation proteins set their pathways’ activating capacities, thus influencing susceptibility to diseases involving complement and coagulation activation, as well as fibrinolytic and anticoagulant pathway activity. In addition, the molecular crosstalk of these evolutionarily linked systems may contribute to concurrent activation and amplification, which may further aggravate pathology. Relevant molecular protein crosstalk of complement and coagulation proteins identified in Table 1(B) and their functional effect on complement and coagulation activity is summarized in Table 2.

**Table 1:** (A) Topmost implicated plasma proteins of the coagulation and complement pathways implicated in longitudinal studies observing conversion to psychotic disorder, or psychotic experiences, and clinical high-risk (CHR) transition to psychotic disorder, as well as case-control comparison for schizophrenia and psychosis spectrum disorders. (B) Overview of topmost implicated plasma proteins of the coagulation and complement pathways identified in two or more studies.

(A)							
Reference	No. of samples	Sample	Disease	Age range	Method	Complement	Coagulation
Transition studies—psychosis							
English et al. [59]*	37 PD, 38 no PD and 40 PE, 66 no PE	Age 12, blood. Comparison of age 18 PD vs non-PD	Psychotic disorder	12	LC–MS/MS	C1R (↑), C1S (↓), CFD (↑), C6 (↑), C7 (↑), C4BP (↓), CFH (↑), CFI (↑), CLU (↑), VTN (↑), IGHM (↓)	FXII (↑), FXI (↑), FIX (↑), FII (↑), FV (↑), FXIII (↑), PLG (↑), SERPINF2 (↑), A2M (↓)
Föcking et al. [60]*	64 PE, 67 no PE	Age 12, blood. Comparison of age 18 PE vs non-PE	Psychotic experiences	12	LC–MS/MS	C1RL (↑), C5 (↑), C8 (↑), C4BP (↓), CFH (↑), VTN (↑), IGHM (↓), IGG (↓)	PLG (↑), A2M (↓)
Madrid-Gambin et al. [61]	48 PE, 67 no PE	Comparison of blood at age 12 against PE at age 18	Psychotic experiences	12	Targeted proteomics (DIA)	VTN (↑)	F11 (↑), HC2 (↑), PLG (↑), SERPINF2 (↑)
Perkins et al. [40]	32 CHR psychosis, 35 HC, 40 CHR no psychosis	Transition vs nontransition	Clinical high risk for psychosis	12–35	Multianalyte profiling, immunoassay	VTN (↑)	FVII (↑), vWF (↑), A2M (↑)
Mongan et al. [10]*	49 transition to psychosis and 84 no transition, 61 PE, 61 HC	Blood, Transition vs nontransition	Schizophrenia (SZ)	18–27 and 12	LC–MS/MS	C1QA (↑), C1QB (↑), C1R (↑), C1S (↑), C1RL (↑), C2 (↑), C3 (↑), C4A (↑), C4B (↑), C5 (↓), C6 (↑), C7 (↑), C8A (↑), C8B (↓), C9 (↓), CFB (↑), CFHR1 (↑), CFHR2 (↑), CFHR5 (↑), CLUS (↑), CFI (↑), CFH (↑), C4BPA (↓), FCN3 (↓), VTN (↓), IGHM (↓)	A2M (↓), F2 (↑), F9 (↑), F10 (↑), F11 (↑), F12 (↑), F13A (↓), F13B (↑), PLG (↑), SERPING1 (↓), SERPINA1 (↓), SERPINA5 (↓), SERPINA10 (↓), PROZ (↓), HC2 (↓), PROC (↓), PROS (↓), SERPINC1 (↑), SERPIND1 (↑)
Case–control studies—psychosis							
Chan et al. [58]	127 first onset SZ, 204 HC	Control vs FEP	SZ	18–49	Multianalyte profiling, immunoassay		FVII (↑), vWF (↑), A2M (↑)
Herberth et al. [143]	17 SZ, 17 HC	Control vs FEP	SZ	22–39	Multianalyte profiling, immunoassay		A2M (↓)
Li et al. [56]*	10 SZ, 10 HC and 47 SZ, 53 HC	Blood, case-control	SZ	24–58.8	LC–MS/MS	C4BPB (↓), C8B (↑), IGHM (↑)	F7 (↓), PROS (↓), SERPINA5 (↑)
Jaros et al. [26]*	20 SZ, 20 HC	Blood, case-control	SZ	22–41.4	Immobilized metal ion affinity chromatography (IMAC) combined with LC–MS/MS	C4BPA (↑), C6 (↑), CFB (↑), FCN3 (↑)	
Levin et al. [25]*	22 SZ, 33 HC	Blood, case-control	SZ	18–44	LC–MS/MS	IGHM(↓)	F13B (↓)
Cooper et al. [144]	60 SZ, 77 HC, 892 blood spot samples	Blood, neonatal blood spots	SZ	23.7–43.7	Multiple reaction monitoring mass spectrometry	C4A (↑), C4BPA (↓), C9 (↑), CLUS (↑)	
Walss-Bass et al. [145]	60 SZ, 20 HC	Blood	SZ	41.1–43.7	X-aptamer technology	C4A (↑)	
Ramsey et al. [146]	133 SZ, 133 HC	Blood, female vs male	SZ	16.5–49.6	Multianalyte profiling, immunoassay	C3 (↑)	F7 (↓), SERPINA1 (↑)
Moriyana et al. [147]*	6 SZ, 6 HC	Umbilical arterial serum	SZ	22.8–38.3	LC–MS/MS	C1QB (↑), C1QC (↑), C1R (↑), C1S (↑), C2 (↑), C3 (↑), C4A (↑), C4B (↑), C5 (↑), C7 (↓), C9 (↑), CFB (↑), CFI (↑), C6 (↑), CLU (↑), VTN (↑), IGHM (↑)	F2 (↑), F10 (↑), F12 (↑), F13B (↑), KLKB1 (↑), SERPINA5 (↑), SERPINC1 (↑), SERPIND1 (↑), HC2 (↑), SERPINF2 (↑), SERPING1 (↑),
Jiang et al. [148]*	20 SZ, 10 HC plus 40 SZ, 40 HC	Leukocyte profiling	First-episode SZ	17.3–33.3	Proteomic signatures	C1QBP (↑), C1QC (↑), C1R (↑), C4B (↑), C4BPA (↑), C6 (↑), C8B (↓), CD59 (↑), CFB (↑), CFD (↑), CFI (↑), CFH (↑), CR1 (↑)	
Gupta et al. [149]	2 SZ, 2 HC	Cerebrospinal fluid	SZ	23–28 (SZ) and 53–60 (HC)	Proteomics, iTRAQ		A2M (↓)
Velasquez et al. [150]	12 SZ, 8 HC	Case-control, brain samples of mitochondria (MIT), crude nuclear fraction (NUC), and cytoplasm (CYT)	SZ	Not stated	Quantitative proteomics, using iTRAQ labeling and SRM	C3 (↑)	
Case–control studies—psychosis spectrum							
Domenici et al. [55]	245 MDD, 229 SZ, 254 HC	Blood, case-control	Major depressive Disorder (MDD) and SZ	27.1–67.5	Multianalyte profiling, immunoassay	C3 (↑)	A2M (↑), F7 (↑), SERPINA1 (↑)
Yang et al. [151]	24 MDD, 12 HC and 98 MDD, 49 HC	Blood, case-control	Major depressive Disorder (MDD), suicide attempters and nonattempters	16–46.5	2-DE-MALDI-TOF/TOF MS and iTRAQ-LC-MS/MS, western blots and ELISAQ	CFB(↑)	F7 (↑), F10 (↓), SERPINA1 (↑)
Turck et al. [152]	39 MDD, 24 responders, 15 nonresponders	Antidepressant treatment	MDD	27.4–64.6		C7 (↓), CFHR1 (↑), CFHR2 (↑), CFHR5 (↑)	F5 (↑), F10 (↓), FGA (↑), FGB (↑), SERPING1 (↓)
Stelzhammer et al. [153]	40 MDD, 63 HC	Depression, case-control, drugnaive	MDD	26.4–53.8	LC–MS/MS	C4B (↑)	
Gui et al. [154]	20 MDD, 20 HC	Blood	MDD	18–60	iTRAQ-based quantitative proteomics (and metabolomics)	CFH (↓)	
de Jesus et al. [155]	14 BPD, 12 HC, 23 SZ, other PD:4	Serum	Bipolar Disorder (BPD), SZ, other Psychotic Disorders	23–55	2D-DiGE	C4A (↑)	
Haenisch et al. [156]	17 BPD, 46 HC	Blood, case-control	BPD	21–47	Multianalyte profiling, immunoassay	C3 (↑)	

(B)							
Pathway	Nested population-based studies (ALSPAC)	Clinical high risk (CHR) – transition (T) vs non-transition (NT) studies	FEP and schizophrenia case-control studies	Other psychosis spectrum case-control studies			
Complement (↑)	**CFH, VTN**	**CFH, VTN** C1Q, C1R, CFI, C5, C7, C8	**C4-B, CFB, C6** C1Q, C1R, C3, CFI, C9, CLU	C3			
Complement (↓)	**C4BP, IGM**	**C4BP, IGM**					
Coagulation (↑)	**FXI, PLG** SERPINF2	**FXI, PLG** FXII, FIX, FII	FXI, PLG FXII, FIX, FII, SERPINF2	FVII, SERPINA1			
Coagulation (↓)	A2M	FXI, PLG FXII, FIX, FII		FX			

In (A), topmost proteins are selected by significance (*p* < 0.05) of expression level fold changes. Studies that conducted comprehensive pathway analyses are labeled with asterisk (*). Upregulation (↑) and downregulation (↓) are indicated for each protein—shown with corresponding gene name.

In (B), overview of protein markers is depicted for longitudinal conversion to psychotic disorder and clinical high risk (CHR)—transition (T) vs nontransition (NT) studies, first-episode psychosis (FEP) and schizophrenia case–control studies, and other psychosis spectrum disorder case–control studies. Longitudinal studies of a general population observing conversion to psychotic experiences and psychotic disorder in the ALSPAC cohort are shown in a separate column. Fold change direction shown for upregulation (↑) and downregulation (↓) is indicated for each protein- shown with corresponding gene name. Only studies that showed consistent fold change direction were included. Proteins marked in bold were found to be altered in >2 studies.

*A2M* alpha-2-macroglobulin, *ADAMTS13* a disintegrin and metalloproteinase with a thrombospondin type 1 motif, member 13 also known as von Willebrand factor-cleaving protease (VWFCP), *C1Q* complement component 1q, *C1R* complement component 1r, *C1RL* complement component 1r Like, *C1S* complement component 1s, *C4BP* complement 4 binding protein, *C5* complement C5, *C6* complement C6, *C7* complement C7, *C8* complement C8, *C8A* complement C8A, *CFB* complement factor B, *CFD* complement factor D, *CFH* complement factor H, *CFI* complement factor I, *CLU* clusterin, *FIC3* ficolin 3, *FII* prothrombin, *FIX* factor IX, *FVII* factor VII, *FXI* factor XI, *FXII* factor XII, *FXIII* factor XIII, *IGG* immunglobulin G, *IGHM* immunoglobulin heavy constant Mu, *IL10* interleukin 10, *IL13* interleukin 13, *IL15* interleukin 15, *IL8* interleukin 8, *PLG* plasminogen, *PROS* vitamin K-dependent protein S, *PROZ* vitamin K-dependent protein Z, *SERPINA7* serpin peptidase inhibitor, clade A member 7, *SERPIND1* serpin family D member 1, *SERPINF2* serpin family F member 2, *SERPING1* plasma protease C1 inhibitor, *VTN* vitronectin, *vWF* van Willebrand factor.

**Table 2:** Overview of molecular crosstalk identified in the topmost implicated plasma proteins of the coagulation and complement pathways.

Coagulation	Complement	Effect on Complement	Effect on Coagulation	Reference
**FXII (↑)**	**C1Q, C1R (↑)**	FXII activates C1 complex	C1Q inhibits activation of FXII in vitro Platelet activation	[157–159]
**FII, FIX, FXI (↑)**	**C3 (↑)**	FIIa, FIXa, and FXIa cleave C3→C3a and C5→C5a	C3a and C5a increase platelet activation C5a increases tissue factor activity C5a increases expression of PAI-1 on mast cells	[160–163]
**FX (↓)**	**C3, C5 (↑)**	FXa cleaves C3 into C3a and C5 into C5a		[160]
**PLG (↑)**	**C5 (↑)**	PLN cleaves C5 into C5a		[141]
**PLG (↑)**	C4BP (↓)		C4BP binds PLG and increases activation of plasminogen (PLG) to plasmin (PLN) C4BP binds PROS resulting decreased cofactor function of protein S for activated protein C	[164, 165]
**FXI, FXII (↑)**	SERPING1		SERPING1 inhibits FXIa and FXIIa	[166]
SERPING1	**C1R (↑)**	SERPING1 inhibits C1R		[167]
**A2M (↓)**	**Thrombin, FXa, PLG (↑)**	A2M, C3, and C4 are structurally similar and evolutionarily related	A2M is a protease inhibitor of thrombin, FXa, plasmin	[168–170]
Platelets	**C7**, **C8**, **C9**	C5b6789 (C5b-9) forms the lytic membrane-attack complex	C5b6789 (C5b-9) affects: Platelet activation; Increased binding of coagulation factors Va and Xa; Increased release of factor V from platelet alpha-granules; Induces endothelial cells to secrete von Willebrand factor; C7 binding interaction with PLG enhances tPA-mediated PLG activation	[164, 171–173]
vWF, TM	**CFH (↑)**	CFH and VWF binding interaction enhances CFH cofactor activity and VWF-mediated platelet aggregation CFH and TM binding interaction enhances CFH cofactor activity		[174–176]
	**CLU (↑)**	CLU binds to C5b-7 and inhibits generation of C5b-9		[177]
Thrombin–antithrombin	**VTN (↑)**	VNT binds to C5b-7 and inhibits C9 polymerization	VTN binds to the thrombin–antithrombin complex (TAT)	[178–180]
Protein S	**C4BP**	C4BP interaction with protein S has no effect on the inhibition of complement activation	C4BP can bind anticoagulant protein S, resulting in a decreased cofactor function of protein S for activated protein C	[181]

Fold change direction shown for upregulation (↑) and downregulation (↓) is indicated for each protein—shown with corresponding gene name. Proteins identified are shown in bold.

A number of studies (Table 1(B)) found significantly altered levels in crosstalk proteins, which potentially impact the activation, amplification, and regulation of both proteolytic systems. Key crosstalk interactions appear to affect generation of complement opsonins and chemotactic effector molecules and activation of coagulation pathways.

## Plasma protein changes in psychosis spectrum disorders

A growing body of literature supports the significant contribution of (neuro)inflammation in the pathogenesis of psychotic disorders [51–54]. Several studies demonstrate changes in plasma protein levels, years before the onset of psychotic experiences or psychotic disorder [10, 40, 55–60]. These highlight the importance of longitudinal studies to identify potential pathophysiological alterations, such as those observed in relation to complement and coagulation pathways [10, 59, 60]. Table 1(A) summarizes the findings for complement and coagulation markers from studies describing altered blood proteins relevant for progression to psychotic disorders, as seen in longitudinal studies of the general population with conversion to psychotic experiences or psychotic disorder as well as CHR individuals transitioning to psychotic disorder. These are compared to markers identified for first-episode psychosis (FEP) and schizophrenia as well as other psychosis spectrum disorders, as shown in Table 1(A).

This breadth of data implicates dysregulation of the complement and coagulation pathways (see Supplementary Table 1 for protein function) suggesting a plausible mechanism, observed years before transition to psychotic experiences [60] or psychotic disorder [59], which was validated in CHR individuals who transitioned to psychotic disorder versus those who did not [10, 40, 58]. There is some observed overlap in protein markers between transition studies and case–control studies assessing FEP or schizophrenia, which further solidifies the relevance of these markers to psychosis. Case–control studies do appear to highlight differences in blood biomarkers (Table 1), however, it is unclear whether these are due to differential study design or reflect pathophysiological changes that are different prior to onset of psychosis that become quiescent in the later stages of CNS manifestation.

It is noteworthy that only a small number of studies use comprehensive analyses such as discovery proteomics to assess the entire complement and coagulation pathways as indicated in Table 1(A). While some studies provide limited data, the study summary (Table 1(B)) clearly shows a robust identification of complement and coagulation biomarkers across all longitudinal studies (complement: high CFH and VTN and low C4BP and IGM; coagulation: high FXI, PLG, and low A2M). This consistency may be attributed to the fact that three out of five studies were conducted in the ALSPAC cohort, a longitudinal study of the general population. In this study, mostly well children were observed, some of whom went on to have psychotic experiences [59–61], and distinct criteria for conversion to psychotic experiences or psychotic disorder were compared. Interestingly, protease inhibitor A2M was consistently downregulated across all conversion and transition studies. A2M was also identified as a marker in schizophrenia case-control studies, albeit not consistently down- or upregulated. We hypothesize that this may reflect differences in clinical stage of illness or possibly the influences of different study design. Table 1(B) further highlights overlapping complement and coagulation proteins, identified for both, longitudinal general population studies with conversion to psychosis and CHR-transition studies, as well as case-control studies (complement: C1Q and C1R; coagulation: FXI and PLG), but also highlights some clear differences that may be a result of distinct study designs or pathophysiological mechanisms during the development of psychosis versus endpoint FEP. In addition, studies involving other psychosis spectrum disorders identified distinct complement and coagulation markers (Table 1(A)), although a lack of comprehensive proteomics discovery studies in these phenotypes discourage speculation as to the relevance of specific markers at this stage.

**Fig. 1:**
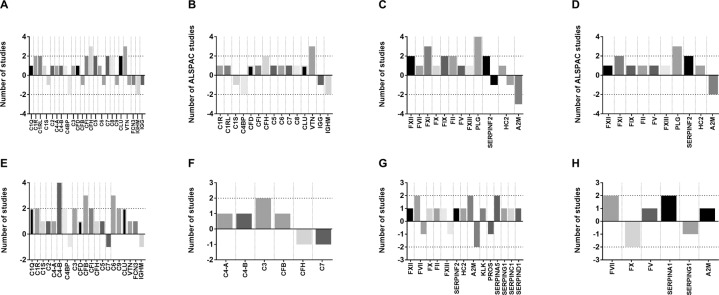
Overview of topmost implicated plasma proteins of the complement and coagulation pathways identified in two or more studies. Number of studies showing altered complement proteins in **A** longitudinal conversion and CHR-transition studies, **B** longitudinal general population studies of the ALSPAC cohort, **C** case-control psychosis studies, and **D** case-control psychosis spectrum studies. Number of studies showing altered coagulation proteins in **E** longitudinal conversion and CHR-transition studies, **F** longitudinal general population studies of the ALSPAC cohort, **G** case-control psychosis studies, and **H** case–control psychosis spectrum studies. Positive or negative depiction indicates direction of fold change found in each study, e.g., a mention of −2 means that two studies found this protein to be downregulated, threshold indicated as two studies.

The topmost complement and coagulation proteins consistently identified across all studies are shown in Fig. 1. Overall, there is a distinctive pattern seen with robust and repeated findings of altered complement and coagulation protein levels in conversion or transition to psychosis and case-control studies. In the longitudinal studies, we observe an upregulation of complement and coagulation components and downregulation of key regulatory molecules, which is not reflected in the cross-sectional case-control studies.

We propose a novel mechanistic hypothesis whereby complement and coagulation protein upregulation results in enhanced activating capacity of the complement and coagulation pathways. Both systems, once triggered, are able to generate effector molecules (such as chemotactic complement anaphylatoxins C3a and C5a) with functions in the inflammatory response.

Therefore, both systems are less likely to reestablish homeostasis post challenge, leading to enhanced complement and coagulation activity and initiation of pathological mechanisms, which advance progression to psychosis. The discrepancies between cross-sectional and longitudinal studies may indicate temporal changes in complement and coagulation protein alterations according to illness stage or simply the lack of comprehensive discovery case-control studies.

To unravel the pathophysiological mechanisms, it is necessary to view the complement and coagulation pathways as integrated and interlinked defense systems that are upregulated in response to cytokine stimuli of the acute phase response to infection or injury. Given the CNS manifestations of psychotic disorder, it is also important to consider their joint effect on the blood-brain barrier (BBB) and in the brain. Here, we briefly review their physiological roles in the blood and the brain.

## Complement and coagulation proteins affect BBB integrity

BBB pathology is recognized as a central factor in the development of many neurological disorders [62]. BBB dysfunction in psychosis might be relevant to many aspects of disrupted neuronal and synaptic function. While it is not clear whether BBB changes are the cause or consequence of neuropathology [63], it is possible that these drive each other, contributing to disease progression. BBB integrity and function [64] disruption, due to systemic inflammation [65] or infection [66], can occur through cytokines affecting BBB integrity [65], as well as CNS functions [67]. For instance, complement C5a-receptor interactions modulate cytokine generation [68] and receptor signaling increases BBB permeability in neuroinflammatory disease [69]. Coagulation proteases thrombin and activated protein C can affect brain pathophysiology by interfering with synapse homeostasis [70] and BBB function [71]. While the plasminogen activation system is key to fibrinolysis [72], in the brain it performs nonfibrinolytic functions [73]. For instance, tissue plasminogen activator (tPA) induces the conversion of plasminogen to plasmin, and promotes BBB permeability via both plasmin‐independent and plasmin‐dependent pathways [73, 74]. Low plasmin activity has been observed in schizophrenia patients [27], as well as low tPA-inhibitor, PAI-1 [75].

While BBB dysfunction is often secondary to the primary insult, it has been suggested as a primary cause in multiple sclerosis, epilepsy, and Alzheimer’s disease [76]. BBB components can actively promote neuroinflammation [76], and BBB disruption may facilitate plasma proteins to enter the brain thereby modulating neuroinflammatory responses [77] and upsetting the fine balance of complement and coagulation components expressed in the brain.

## Complement and coagulation in the brain

We provide evidence for the role of systemic alterations of complement and coagulation proteins in the blood. While these are primarily expressed in the liver, all complement components can be locally produced in the brain [78]. The local role of the complement and coagulation systems in the brain in terms of psychosis pathophysiology is not completely established.

Local complement synthesis, modulated by proinflammatory cytokines, was confirmed in the context of Alzheimer’s disease in human astrocytes and microglia, and in this context serves mainly to aid clearance of apoptotic cells or debris [79] with subsequently identified CNS roles in synaptic function [80]. During fetal development, complement proteins contribute to diverse neurodevelopmental processes, while dysregulation can alter the correct balance manifesting in neurodevelopmental disorders [43]. Complement classical pathway (CP) components have been described to aid synaptic elimination [81] and microglial-mediated synaptic pruning [82]. Excessive synaptic pruning on the other hand has not only been associated with developmental and brain diseases but also with normal postnatal CNS development [83]. Therefore, altered complement expression and dysregulation in the brain could result in enhanced synapse pruning, neuronal injury, and neurotoxicity, as seen in neuroinflammatory and neurodegenerative diseases [84].

Coagulation components, in particular the plasminogen activation system, influence numerous aspects of brain function [74, 85], neuroinflammatory processes [86], and neurodegeneration [87], with evidence that fibrin increases axonal damage, microglial activation, and clinical severity in neuroinflammatory disease [88]. The importance of the fibrinolytic system and plasminogen activation in fibrin removal is well characterized, and there is evidence that the plasminogen activation system contributes to brain function [72, 74, 89]. In addition, fibrinogen leakage upon BBB disruption leads to activation of innate immunity in the CNS [90] and fibrin(ogen) deposits in the CNS has a pathogenic role in neurodegenerative and other diseases [91]. Fibrinogen can drive neuroinflammatory responses through CD11b/CD18 (complement receptor 3) [83, 92] and induces microglial-mediated synaptic elimination, while deposition of different coagulation factors may trigger exacerbation of inflammation [93]. For instance, coagulation factors TF, thrombin, or fibrinogen are described as potential drivers of inflammation in disease models [94–98].

These integral roles of complement and coagulation components in neurodevelopment and CNS function, alongside the observed complement and coagulation changes, link systemic changes in blood with changes in synaptic plasticity, vulnerability to neuroinflammation, and neurodegeneration in the brain. However, it is not clear whether synaptic changes observed in psychosis [99, 100] occur as a result of systemic alterations, synergistic changes in the blood and brain or whether they represent potentially independent pathophysiological mechanisms.

## Role of complement and coagulation during development

Novel findings indicate that progression to future psychotic experiences and disorder are associated with the presence of an early inflammatory phenotype driven by complement and coagulation pathway-related mechanisms [10, 59, 60]. This enhanced inflammatory-complement and coagulant tone could be triggered by prenatal infection [101, 102], a long-known risk factor for schizophrenia. While normal pregnancy is characterized by an enhanced innate and suppressed adaptive immune response [103], increased plasma levels of complement [104, 105] and acute phase proteins [106] could lead to enhanced complement activity. Complement activation and dysregulation contributes to multiple adverse pregnancy outcomes, and highlights the importance of complement regulation at the fetal-maternal interface [107] and a possible association with maternal immune activation [108–110].

Longitudinal studies, from in utero to early childhood, allow examination of associations between the prenatal environment, brain development, and later behavioral alterations [109]. These could clarify whether the observed complement and coagulation changes are present at birth or a result of maternal immune activation [108–110] and could extend into adolescence. While studies have identified altered cytokine expression in association with risk for psychosis [111], these studies have not yet addressed the involvement of the complement and coagulation pathways in these phenotypes at early developmental stages.

A notable potential postnatal influencing factor is that of childhood adversity. Individuals with a history of childhood adversity have higher levels of circulating markers of acute and chronic inflammation [112, 113] and there is evidence of increased hypothalamic-pituitary-adrenal axis activation and blunted response to stress in FEP [114]. Exposure to stress is a trigger of inflammation associated with neuroinflammation and neurodegenerative disease [115]. Recent evidence indicates that stress can activate the inflammatory response in the brain as well as peripherally [116, 117]. We have observed dysregulated expression of a number of complement proteins in mice exposed to chronic social stress, which were altered in the same direction as seen in subjects progressing to psychotic experiences [60]. This suggests that complement level alterations may also reflect exposure to stress.

Complement is integral to the early immune response against microbial infection [45], which is a recognized risk factor for psychotic disorders. For instance, maternal infection with influenza early in gestation or toxoplasmosis are associated with an increased risk of schizophrenia for the offspring [101, 118], as well as postnatal infections later in life contributing to risk [52]. While an enhanced inflammatory-complement phenotype should support resolution of infection, if unable to reestablish homeostasis post challenge, this could then contribute to chronic inflammation [119].

## Complement and coagulation alterations potentially represent a key biological vulnerability, or predisposition, in the progression to psychosis

Several factors likely contribute to the development and eventual CNS manifestation of psychotic disorder and the broader psychosis spectrum. The two-hit hypothesis for schizophrenia suggests that a genetic and prenatal environmental “first-hit” affects brain development, and establishes increased vulnerability to a “second-hit” that may occur later in life [11–13].

**Fig. 2:**
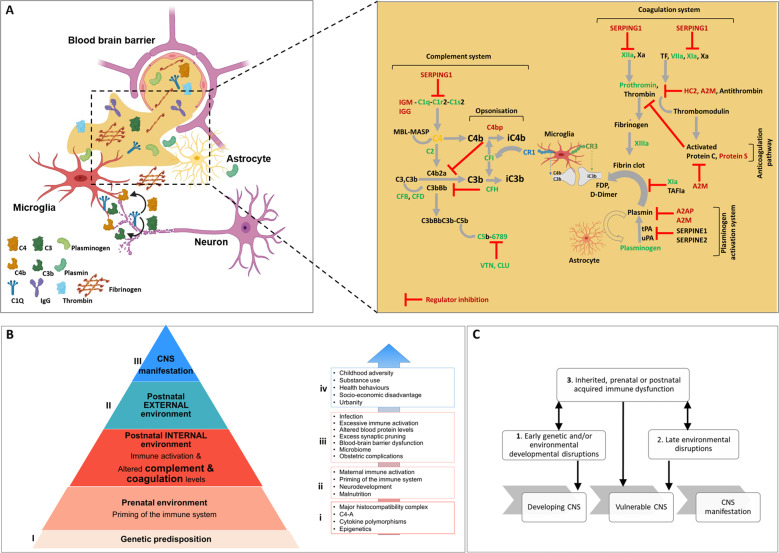
Mechanism by which infection and inflammation (inherited and acquired risk) influence the progression to psychosis. **A** Complement and coagulation proteins and pathways show upregulated components in green (proteomics studies) or blue (transcriptomic studies). Downregulated components are shown in red. Regulatory interactions are highlighted with an inhibition arc colored in red **├**. Genetic variant is shown in orange. A blood-brain barrier (BBB) cross-section with complement and coagulation proteins at the neurovascular interface and extravasation into the central nervous system (CNS) following disintegration of the BBB. Activation of the coagulation and plasminogen activation and complement system promoting inflammation and microglia-mediated cellular damage. **B** The pyramid describes the cumulative risk factors in the progression toward psychosis spectrum disorders illustrating the integrated impact of genetic predisposition and prenatal environment (priming of the immune system), postnatal internal environment (immune activation, altered complement, and coagulation levels), postnatal external environment, leading to central nervous system (CNS) manifestation. Notably, each risk factor is not considered in isolation but as a significant contributing factor and each of the “hits” may have an additive effect on the progression to psychotic disorder. Points of risk assessment and intervention: (I) Risk assessment and stratification, e.g., prenatal environment and genetic risk association, (II) stratification, e.g., clinical high risk (CHR), proposed predicative blood biomarkers and intervention, e.g., cognitive behavioral therapy (CBT), Omega-3 supplementation and proposed anti-inflammatory (complement) and anticoagulant therapeutics. (III) Intervention, e.g., cognitive behavioral therapy (CBT) and antipsychotic drugs. The box and arrow breaks down the individual components as bullet points associated with (i) genetic predisposition, (ii) prenatal environment, (iii) postnatal internal environment, and (iv) postnatal external environment. **C** The two-hit hypothesis of schizophrenia states that early genetic and/or environmental developmental disruptions (“first-hit”) to the developing central nervous system (CNS) increase the vulnerability of the individual to subsequent, late environmental disruptions (“second-hit”), leading to the development of CNS manifestation. The multiple-hit theory introduces another hit of inherited, prenatal, or postnatal acquired immune dysfunction that sets complement and coagulation pathway activating capacity and regulatory ability integral to development of psychotic disorder (adapted from [12]). A2M alpha-2-macroglobulin, APC activated protein C, AT antithrombin, C1Q complement component 1q, C1r complement C1r subcomponent, C1S complement component 1s, C2 complement C2, C3 complement C3, C3b is the larger of two elements formed by the cleavage of complement component 3, opsonins, C3bBb complement C3 convertase, C4 complement C4, C4b is the larger of two elements formed by the cleavage of complement component 4, opsonin, C4b2a complement C3 convertase, C5b6789 membrane-attack complex, CFB complement factor B, CFD complement factor D, CFH complement factor H, CLU clusterin, D-dimer a fibrin degradation product, FDP fibrin degradation products, FII prothrombin; HC2 heparin cofactor II or SERPIND1, iC3b inactive component 3b, opsonin, IGG immunoglobulin G, IGM immunglobulin M, MASP mannose-associated serine protease, MBL mannose-binding lectin, PAI-1 plasminogen activator inhibitor-1, PROS vitamin K-dependent protein S, SERPING1 plasma protease C1 inhibitor, TF tissue factor, tPA tissue plasminogen activator, VIIa plasma factor VIIa, VTN vitronectin, Xa factor Xa, XIa factor XIa, XIIa factor XIIa, XIIIa factor XIIIa, an enzyme of the blood coagulation system that crosslinks fibrin.

We put forward a novel extension of the two-hit hypothesis; we propose that altered complement and coagulation protein levels set their pathways activating capacity, thus influencing susceptibility to immune activation (Fig. 2). This “at-the-ready” state can lead to increased activation when triggered and- as a result of low regulatory components- leads to amplification of these systems. Importantly, lack of key regulatory proteins in these pathways cause dysregulation and pan-pathway crosstalk further amplifies the generation of effector molecules. While under normal circumstances, activation and regulation of these systems are finely balanced and work to reestablish homeostasis in response to an inflammatory insult [42], we propose that the observed alterations may represent a biological vulnerability, or predisposition, in the progression to psychosis. We hypothesize that psychosis is a multiple-hit pathology where several triggering events in a biologically vulnerable individual might lead to psychosis. The observed alterations in complement and coagulation components may represent another “hit” adding to many other genetic or environmental risk factors. We define this distinct vulnerability or “hit” as “dysregulated complement and coagulation activation, and dysfunction of the fibrinolytic and anticoagulant pathways.” A loss of regulatory components could lead to enhanced and persistent complement and coagulation dysfunction. Further research is required to determine whether the enhanced activating capacity or the lack of regulation is a key pathophysiological factor in the development of psychotic disorder and if loss of regulatory components is a result of inherited or acquired deficiency. Overall, complement deficiencies are rare primary immunodeficiency disorders, poorly characterized clinically as they have been difficult to detect, and are probably underdiagnosed in clinical practice [120]. Deficiencies of complement components can be inherited or acquired, partial or complete, and increase susceptibility to infections. Infectious diseases are well known to provoke psychiatric symptoms such as viral infection with herpes simplex virus [121, 122], measles [123] and neuropsychiatric associations with human immunodeficiency virus [124], respiratory virus infections including influenza [125], and most recently SARS-CoV-2 [126–128].

Susceptibility to infections, when the delicate balance between complement and coagulation homeostasis is altered, may contribute to enhanced vulnerability to psychosis as a result of multiple risk factors that influence the development of psychosis, and may have an additive effect (“hit”) on the progression to psychotic disorder. For instance, genetic [16] or early environmental [107, 109] psychosis risk factors are kept in check, unless complement or coagulation pathways are triggered, through, e.g., environmental risk factors [129], or infection [118, 130, 131].

We hypothesize that altered complement and coagulation component levels set their activating capacity. Once triggered (e.g., infection) these cascades generate effector molecules (e.g., opsonins, anaphylatoxins, active serine proteases, fibrin clot). We propose that dysregulation (lack or loss of regulatory components) is the key underlying mechanism driving the amplification of these pathways leading to immune activation and inability to reestablish homeostasis. Peripheral dysregulated complement and coagulation activation leads to cellular and possibly vascular damage, which over time, contributes to BBB leakiness, potential loss of transport regulation, and neuroinflammation [69] and eventual loss of BBB integrity. Plasma extravasation into the brain and potentially altered local expression of complement and coagulation proteins upset the balance of physiological mechanisms (e.g., synaptic plasticity). In addition to extravasation of activated complement and coagulation components, their expression in the brain could be also altered, albeit not necessarily similarly, thus setting activating and regulatory capacity in the brain. Increased complement opsonization could affect complement pathway activating capacity and opsonin-mediated synaptic pruning. The extravasation of coagulation components into the CNS can drive neuroinflammatory responses through deposition of different coagulation factors. Fibrinogen can induce further microglial-mediated synaptic elimination and coagulation serine proteases are further able to generate complement effector molecules (Fig. 2A).

While normal activity of neuroinflammation acts mainly to restore brain homeostasis, prenatal genetic and environmental “first-hit” vulnerability may contribute to the development of CNS manifestation. The pyramid (Fig. 2B) describes the cumulative risk factors in the progression toward psychosis spectrum disorders illustrating the integrated impact of genetic predisposition and prenatal environment (priming of the immune system), postnatal internal environment (immune activation, altered complement, and coagulation levels), and postnatal external environment, leading to CNS manifestation.

Notably, each risk factor is not considered in isolation but as a significant contributing factor and each of the “hits” may have an additive effect on the progression to psychotic disorder. Increased immune activation may contribute to these or even present as a distinct “hit” of inherited, prenatal or postnatal acquired immune dysfunction that sets complement and coagulation pathway activating capacity and regulatory ability and is integral to development of psychotic disorder (Fig. 2C).

It remains a question of debate and further research whether complement and coagulation dysfunction can be seen as a distinct “hit” or is better described as a phase of the immune activation (“second-hit”).

These risk factors in the progression to psychosis may be counterbalanced by a number of measures. Therapeutic interventions at this stage may contribute to reduced neuroinflammation and maintenance of the BBB to prevent progression to psychotic disorder. There is some evidence that therapies targeting inflammatory and immune pathways show efficacy in treatment trials for psychosis. These include nonsteroidal anti-inflammatory drugs as adjuncts to antipsychotics in patients with schizophrenia [132] or other drugs such as minocycline [133], statins, and omega-3 fatty acids [134].

Complement therapeutics are emerging for other disease phenotypes characterized by complement deficiency and dysregulation [135, 136], any association with psychotic symptoms have not been reported yet. Interestingly, anticoagulant therapy has been associated with remission of psychotic symptoms [137] and therefore, anticoagulants could be considered in future research as adjunctive treatments, for instance, target-specific anticoagulants [138]. The efficacy of fibrin-targeting therapeutics needs further investigation [139] considering fibrinogen extravasation into the CNS as a mediator of neurodegeneration [90].

## Future directions

Outstanding questions remain. For example, it is unknown whether the altered blood proteome observed in psychosis arises from dysfunctional hepatic or nonhepatic cytokine signaling, acute phase response dysfunction, genetic risk factors, or consumption-mediated deficiency of key regulatory complement and coagulation components. Further investigation is needed to determine whether blood plasma protein expression is correlated with the brain, noting that all key components significantly altered in blood proteome studies are also expressed in the brain and contribute to key CNS functions [78, 80, 81, 140].

To understand the mechanisms of immune activation, we need to investigate the underlying inherited, prenatal or postnatal acquired immune dysfunction mechanisms and their key triggers that cause complement and coagulation dysfunction in psychosis and whether the pathophysiological roles of the complement and/or coagulation systems are distinct “hits” in the progression to psychosis. Therefore, we require further knowledge of the (i) mechanisms that lead to differential protein synthesis (congenital deficiency) or consumption-associated acquired deficiency, (ii) distinct triggers of these proteolytic cascades, (iii) genetic or environmental factors that contribute to dysregulation and altered activating capacity, and (iv) functional consequences of molecular crosstalk.

To confirm whether complement or coagulation deficiency leads to neuropsychiatric disorders, we require the development of mouse models. In line with our findings suggesting that dysregulation of the complement protein pathway reflects past exposure to stress [60], a chronic social stress or an environmental “two-hit” animal model in complement deficient mice could determine whether complement deficiency influences pathophysiology.

Second, it is unclear whether complement and coagulation pathways are dysregulated in the prenatal environment or become established during postnatal development among those who later develop psychotic disorder. Therefore, studies of longitudinal cohorts analyzing the complement and coagulation activation fragments, which are generated when triggering these pathways, are needed and will give an in-depth view of pathway-specific dysregulation in individuals with phenotypes across the psychosis spectrum.

Third, the role of C4 variants [16] (“first-hit”) on the functional effect of C4BP regulating complement C4b-A and -B variants that derives from C4A or C4B needs further investigation. The effect of low plasma concentration of C4BP [59, 60] in conjunction with low protein S on the anticoagulant pathway [28] needs further clarification in these patients. The role of C4BP in the activation of plasminogen [141], due to the association of the plasminogen activation system with schizophrenia [27], merits biochemical investigation of the synergistic impact of this crosstalk interaction on systemic complement and coagulation activity. While C4 and plasminogen/plasmin have been found in the brain, and C4BP was immunohistochemically detected on apoptotic cells in Alzheimer’s brain [142], it remains unclear whether molecular crosstalk or any of the complement and coagulation expression changes that we have observed translate to the psychosis brain.

Lastly, while ours and several other studies have covaried for environmental and other factors, such as BMI, gender, or infection, residual confounding is always possible in observational studies.

## Conclusions

There is now compelling evidence for a key role of complement and coagulation alterations in psychosis spectrum disorders and we propose that these changes lead to increased risk for psychosis. Intriguingly, the evidence may be most robust for dysregulation in early psychosis phenotypes, such as in advance of psychotic experiences among apparently well children, or prior to the onset of psychotic disorder among the high-risk population. The observed pattern of protein changes in these pathways has been consistent and points to the presence of chronic immune activation as a risk factor for psychosis. Critically, while the conceptualization of psychosis as an inflammatory condition and immune activation is not new, our data provide a novel understanding and refocus on the consolidated functions of the complement and coagulation pathways contributing to the progression to psychotic disorder. These pathways may provide objective biomarkers of psychosis, and enable identification of therapeutic targets for early and more effective intervention strategies and treatment.

## Supplementary information

Supplementary material
