# Supplementary material for: Dysregulation of complement and coagulation pathways: emerging mechanisms in the development of psychosis
Source: Mol Psychiatry. 2021 Jul 5;27(1):127–40. doi: 10.1038/s41380-021-01197-9 (PMC8256396; doi:10.1038/s41380-021-01197-9)
Supplement: Supplementary file 1 — Supplementary material [file 41380_2021_1197_MOESM1_ESM.docx]

**Supplementary**

**Supplementary Material 1.**

**Supplementary Table 1.**

**Supplementary Material 1. Risk pyramid describing the progression steps towards psychosis spectrum disorders**: ***(i)*** genetic predisposition; ***(ii)*** prenatal environment, ***(iii)*** postnatal internal environment, and ***(iv)*** postnatal external environment.

**(i)**

Major histocompatibility complex ([1](#_ENREF_1))

C4-A ([2](#_ENREF_2), [3](#_ENREF_3))

Cytokine polymorphism ([4](#_ENREF_4))

Epigenetics ([5](#_ENREF_5))

**(ii)**

Maternal immune activation ([6](#_ENREF_6))

Priming of the immune system ([7](#_ENREF_7))

Neurodevelopment ([8](#_ENREF_8))

Malnutrition ([9](#_ENREF_9))

**(iii)**

Infection ([10](#_ENREF_10))

Excessive immune activation ([11](#_ENREF_11))

Altered blood protein levels ([12](#_ENREF_12))

Excessive synaptic pruning ([13](#_ENREF_13))

Blood brain barrier dysfunction ([14](#_ENREF_14))

Microbiome ([15](#_ENREF_15))

Obstetric complications ([16](#_ENREF_16))

**(iv)**

Childhood adversity ([17](#_ENREF_17))

Substance use ([18](#_ENREF_18))

Health behaviours ([19](#_ENREF_19))

Socio-economic disadvantage ([20](#_ENREF_20))

Urbanicity ([21](#_ENREF_21))

**Supplementary Table 1. Complement and coagulation component function.** Protein ID and its gene ontology (GO) molecular function as well as biological processes were taken from Uniprot (uniprot.org). Proteins listed in alphabetical order.

| **Gene ID** | **Protein** | **Pathway** | **Uniprot ID** | **Function** |
| --- | --- | --- | --- | --- |
| A2M | Alpha-2-Macroglobulin | Other | P01023 | GO - Molecular function  calcium-dependent protein binding  endopeptidase inhibitor activity  enzyme binding  growth factor binding  interleukin-1 binding  interleukin-8 binding  protease binding  serine-type endopeptidase inhibitor activity  signaling receptor binding  tumor necrosis factor binding  GO - Biological processing  blood coagulation, intrinsic pathway, extracellular matrix disassembly negative regulation of complement activation, lectin pathway  platelet degranulation  regulation of small GTPase mediated signal transduction, stem cell differentiation |
| ADAMTS13 | A disintegrin and metalloproteinase with a thrombospondin type 1 motif, member 13 | Coagulation | Q76LX8 | GO - Molecular function  calcium ion binding, integrin binding, metalloendopeptidase activity  metallopeptidase activity, zinc ion binding  GO Biological Process:  cell-matrix adhesion; cellular response to interferon-gamma  cellular response to interleukin-4  cellular response to lipopolysaccharide  cellular response to tumor necrosis factor  glycoprotein metabolic process  integrin-mediated signaling pathway  peptide catabolic process  platelet activation  protein processing  proteolysis |
| C1Q | Complement component 1q | Complement | P02747 | GO - Biological process  complement activation  complement activation, classical pathway  immune response  innate immune response  negative regulation of granulocyte differentiation  negative regulation of macrophage differentiation  regulation of complement activation  synapse pruning |
| C1R | Complement component 1r | Complement | P00736 | GO - Molecular function  calcium ion binding  serine-type endopeptidase activity  serine-type peptidase activity  GO - Biological process  complement activation, classical pathway  immune response  innate immune response  regulation of complement activation  zymogen activation |
| C1RL | Complement component 1r Like | Complement | Q9NZP8 | GO - Molecular function  serine-type endopeptidase activity  GO - Biological process  complement activation, classical pathway  innate immune response, zymogen activation |
| C1S | Complement component 1s | Complement | P09871 | GO - Molecular function  calcium ion binding  identical protein binding  serine-type endopeptidase activity  GO - Biological process  complement activation  complement activation, classical pathway  innate immune response  regulation of complement activation |
| C4BP | Complement 4 binding protein | Complement | P04003 | GO - Molecular function  RNA binding  GO - Biological process  complement activation, classical pathway  innate immune response  negative regulation of complement activation, classical pathway  positive regulation of protein catabolic process  regulation of complement activation  regulation of opsonization  response to symbiotic bacterium |
| C5 | Complement C5 | Complement | P01031 | GO - Molecular function  chemokine activity  endopeptidase inhibitor activity  signaling receptor binding  GO - Biological process  activation of MAPK activity  cell surface receptor signaling pathway  chemotaxis  complement activation, alternative pathway  complement activation, classical pathway  cytolysis  G protein-coupled receptor signaling pathway  inflammatory response  negative regulation of macrophage chemotaxis Source: BHF-UC  positive regulation of chemokine secretion  positive regulation of vascular endothelial growth factor production  regulation of complement activation |
| C6 | Complement C6 | Complement | P13671 | GO - Biological process  complement activation  complement activation, classical pathway  cytolysis  innate immune response  in utero embryonic development  positive regulation of activation of membrane attack complex  positive regulation of angiogenesis  regulation of complement activation |
| C7 | Complement C7 | Complement | P10643 | GO - Biological process  cellular sodium ion homeostasis  complement activation  complement activation, alternative pathway  complement activation, classical pathway  cytolysis  regulation of complement activation |
| C8A | Complement C8A | Complement | P07357 | GO - Molecular function  complement binding  protein-containing complex binding  GO - Biological process  complement activation  complement activation, alternative pathway  complement activation, classical pathway  cytolysis  immune response  regulation of complement activation |
| CFB | Complement factor B | Complement | P00751 | GO - Molecular function  complement binding  serine-type endopeptidase activity  GO - Biological process  complement activation  complement activation, alternative pathway  regulation of complement activation |
| CFD | Complement factor D | Complement | P00746 | GO - Molecular function  serine-type endopeptidase activity  serine-type peptidase activity  GO - Biological process  complement activation  complement activation, alternative pathway  neutrophil degranulation  Notch signaling pathway  platelet degranulation  proteolysis |
| CFH | Complement factor H | Complement | P08603 | GO - Molecular function  heparan sulfate proteoglycan binding  heparin binding  identical protein binding  GO - Biological process  complement activation  complement activation, alternative pathway  regulation of complement activation  regulation of complement-dependent cytotoxicity  viral process |
| CFI | Complement factor I | Complement | P05156 | GO - Molecular function  metal ion binding  scavenger receptor  serine-type endopeptidase activity  GO - Biological process  complement activation, classical pathway  innate immune response  regulation of complement activation  viral process |
| CLU | Clusterin | Complement | P10909 | GO - Molecular function  amyloid-beta binding  chaperone binding  low-density lipoprotein particle receptor binding  misfolded protein binding  protein-containing complex binding  protein heterodimerization activity  signaling receptor binding  tau protein binding  ubiquitin protein ligase binding  unfolded protein binding  GO - Biological process  antimicrobial humoral response  cell  central nervous system myelin maintenance  chaperone-mediated protein complex assembly  chaperone-mediated protein folding  chaperone-mediated protein transport involved in chaperone-mediated autophagy  complement activation  complement activation, classical pathway  immune complex clearance  innate immune response |
| FIC3 | Ficolin 3 | Complement | O75636 | GO - Molecular function  antigen binding  carbohydrate binding  metal ion binding  GO - Biological process  complement activation  complement activation, lectin pathway  defense response to virus  negative regulation of RNA biosynthetic process  negative regulation of viral entry into host cell  recognition of apoptotic cell |
| FII | Factor II, Prothrombin | Coagulation | P00734 | GO - Molecular function  calcium ion binding  enzyme activator activity  growth factor activity  heparin binding  lipopolysaccharide binding  serine-type endopeptidase activity  signaling receptor binding  thrombospondin receptor activity  GO - Biological process  acute-phase response  antimicrobial humoral immune response mediated by antimicrobial peptide  blood coagulation  blood coagulation, intrinsic pathway  cell surface receptor signaling pathway  cellular protein metabolic process  cytolysis by host of symbiont cells  endoplasmic reticulum to Golgi vesicle-mediated transport  fibrinolysis  G protein-coupled receptor signaling pathway  leukocyte migration |
| FIX | Factor IX | Coagulation | P00740 | GO - Molecular function  calcium ion binding  endopeptidase activity  serine-type endopeptidase activity  GO - Biological process  blood coagulation  blood coagulation, intrinsic pathway  endoplasmic reticulum to Golgi vesicle-mediated transport  proteolysis  zymogen activation |
| FVII | Factor VII | Coagulation | P08709 | GO - Molecular function  calcium ion binding  serine-type endopeptidase activity  serine-type peptidase activity  signaling receptor binding  GO - Biological process  animal organ regeneration  blood coagulation  blood coagulation, extrinsic pathway  circadian rhythm  endoplasmic reticulum to Golgi vesicle-mediated transport  positive regulation of blood coagulation  positive regulation of cell migration  positive regulation of leukocyte chemotaxis  positive regulation of platelet-derived growth factor receptor signaling pathway |
| FXI | Factor XI | Coagulation | P03951 | GO - Molecular function  heparin binding  identical protein binding  serine-type aminopeptidase activity  serine-type endopeptidase activity  GO - Biological process  blood coagulation  blood coagulation, intrinsic pathway  plasminogen activation  positive regulation of fibrinolysis |
| FXII | Factor XII | Coagulation | P00748 | GO - Molecular function  calcium ion binding  misfolded protein binding  serine-type endopeptidase activity  GO - Biological process  blood coagulation, intrinsic pathway  Factor XII activation  fibrinolysis  innate immune response  plasma kallikrein-kinin cascade  positive regulation of blood coagulation  positive regulation of fibrinolysis  positive regulation of plasminogen activation  protein autoprocessing  protein processing  proteolysis  response to misfolded protein  zymogen activation |
| FXIII | Factor XIII | Coagulation | P05160 | GO - Biological process  blood coagulation  negative regulation of cellular protein catabolic process |
| IGHM | Immunoglobulin Heavy Constant Mu | Other | P01871 | GO - Molecular function  antigen binding  GO - Biological process  adaptive immune response  antibacterial humoral response  B cell receptor signaling pathway  complement activation, classical pathway  defense response to bacterium  defense response to Gram-negative bacterium  innate immune response  leukocyte migration  phagocytosis, engulfment  phagocytosis, recognition  positive regulation of B cell activation |
| IL10 | Interleukin 10 | Cytokine | P22301 | GO - Molecular function  cytokine activity  growth factor activity  interleukin-10 receptor binding  protein dimerization activity  GO - Biological process  aging  B cell differentiation  branching involved in labyrinthine layer morphogenesis  cellular response to estradiol stimulus  cellular response to hepatocyte growth factor stimulus  cellular response to lipopolysaccharide  cytokine-mediated signaling pathway  cytoplasmic sequestering of NF-kappaB  defense response to bacterium  defense response to protozoan  endothelial cell apoptotic process  hemopoiesis  interleukin-12-mediated signaling pathway |
| IL13 | Interleukin 13 | Cytokine | P35225 | GO - Molecular function  cytokine activity  interleukin-13 receptor binding  GO - Biological process  cellular response to mechanical stimulus  cytokine-mediated signaling pathway  immune response  inflammatory response  macrophage activation  microglial cell activation  negative regulation of complement-dependent cytotoxicity  negative regulation of endothelial cell apoptotic process  negative regulation of inflammatory response  negative regulation of lung ciliated cell differentiation  negative regulation of NAD(P)H oxidase activity  negative regulation of neuron death  negative regulation of transforming growth factor beta production |
| IL15 | Interleukin 15 | Cytokine | P40933 | GO - Molecular function  cytokine activity  cytokine receptor binding  GO - Biological process  aging  cell-cell signaling  cell maturation  cellular response to vitamin D  extrathymic T cell selection  hyaluronan metabolic process  immune response  inflammatory response  interleukin-15-mediated signaling pathway  lymph node development  macrophage differentiation  negative regulation of cold-induced thermogenesis  negative regulation of smooth muscle cell proliferation  neutrophil activation  NK T cell proliferation |
| IL8 | Interleukin 8 | Cytokine | P10145 | GO - Molecular function  chemokine activity  CXCR chemokine receptor binding  interleukin-8 receptor binding  GO - Biological process  angiogenesis  antimicrobial humoral immune response mediated by antimicrobial peptide  calcium-mediated signaling  cell cycle arrest  cellular response to fibroblast growth factor stimulus  cellular response to interleukin-1  cellular response to lipopolysaccharide  cellular response to tumor necrosis factor  chemokine-mediated signaling pathway  chemotaxis  cytokine-mediated signaling pathway |
| PLG | Plasminogen | Coagulation | P00747 | GO - Molecular function  apolipoprotein binding  chaperone binding  endopeptidase activity  enzyme binding  kinase binding  proteasome core complex binding  protein antigen binding  protein domain specific binding  serine-type endopeptidase activity  serine-type peptidase activity  signaling receptor binding  GO - Biological process  blood coagulation  cellular protein metabolic process  extracellular matrix disassembly  fibrinolysis  interaction with symbiont  labyrinthine layer blood vessel development  mononuclear cell migration  muscle cell cellular homeostasis  myoblast differentiation  negative regulation of cell-cell adhesion mediated by cadherin |
| PROS | **Vitamin K-dependent protein S** | Coagulation | P07225 | GO - Molecular function  calcium ion binding  endopeptidase inhibitor activity  GO - Biological process  blood coagulation  endoplasmic reticulum to Golgi vesicle-mediated transport  fibrinolysis  leukocyte migration  platelet degranulation  regulation of complement activation |
| PROZ | Vitamin K-dependent protein Z | Coagulation | P22891 | GO - Molecular function  calcium ion binding  serine-type endopeptidase activity  GO - Biological process  blood coagulation  endoplasmic reticulum to Golgi vesicle-mediated transport |
| SERPINA7 | Serpin Peptidase Inhibitor, Clade A member 7 | Coagulation | P05543 | GO - Molecular function  serine-type endopeptidase inhibitor activity  GO - Biological process  negative regulation of endopeptidase activity  thyroid hormone transport |
| SERPIND1 | Serpin Family D Member 1 | Coagulation  Complement | P05546 | GO - Molecular function  endopeptidase inhibitor activity  heparin binding  serine-type endopeptidase inhibitor activity  GO - Biological process  blood coagulation  cellular protein metabolic process  chemotaxis  negative regulation of endopeptidase activity  post-translational protein modification |
| SERPINF2 | Serpin Family F Member 2 | Coagulation | P08697 | GO - Molecular function  endopeptidase inhibitor activity  protease binding  protein homodimerization activity  serine-type endopeptidase inhibitor activity  GO - Biological process  acute-phase response  blood vessel morphogenesis  collagen fibril organization  fibrinolysis  negative regulation of endopeptidase activity  negative regulation of fibrinolysis  negative regulation of plasminogen activation |
| SERPING1 | **Plasma protease C1 inhibitor** | Coagulation | P05155 | GO - Molecular function  serine-type endopeptidase inhibitor activity  GO - Biological process  aging  blood circulation  blood coagulation, intrinsic pathway  complement activation, classical pathway  fibrinolysis  innate immune response  negative regulation of complement activation, lectin pathway  negative regulation of endopeptidase activity  platelet degranulation  regulation of complement activation |
| VTN | Vitronectin | Complement | P04004 | GO - Molecular function  collagen binding  extracellular matrix binding  extracellular matrix structural constituent  heparin binding  identical protein binding  integrin binding  polysaccharide binding  scavenger receptor activity  GO - Biological process  cell adhesion  cell adhesion mediated by integrin  cell-matrix adhesion  cell migration  endodermal cell differentiation  extracellular matrix organization  immune response  liver regeneration  negative regulation of blood coagulation  negative regulation of endopeptidase activity  oligodendrocyte differentiation |
| vWF | van Willebrand Factor | Coagulation | P04275 | GO - Molecular function  chaperone binding  collagen binding  identical protein binding  immunoglobulin binding  integrin binding  protease binding  protein N-terminus binding  GO - Biological process  blood coagulation  blood coagulation, intrinsic pathway  cell adhesion  cell-substrate adhesion  extracellular matrix organization  hemostasis  platelet activation  platelet degranulation  response to wounding |

1. Biological insights from 108 schizophrenia-associated genetic loci. Nature. 2014;511(7510):421-7.

2. Sekar A, Bialas AR, de Rivera H, Davis A, Hammond TR, Kamitaki N, et al. Schizophrenia risk from complex variation of complement component 4. Nature. 2016;530(7589):177-83.

3. Kamitaki N, Sekar A, Handsaker RE, de Rivera H, Tooley K, Morris DL, et al. Complement genes contribute sex-biased vulnerability in diverse disorders. Nature. 2020.

4. Momtazmanesh S, Zare-Shahabadi A, Rezaei N. Cytokine Alterations in Schizophrenia: An Updated Review. Frontiers in psychiatry. 2019;10:892.

5. Akbarian S. Epigenetic mechanisms in schizophrenia. Dialogues Clin Neurosci. 2014;16(3):405-17.

6. Conway F, Brown AS. Maternal Immune Activation and Related Factors in the Risk of Offspring Psychiatric Disorders. Frontiers in psychiatry. 2019;10:430.

7. Brown AS, Susser ES. Prenatal nutritional deficiency and risk of adult schizophrenia. Schizophr Bull. 2008;34(6):1054-63.

8. Owen MJ, O'Donovan MC, Thapar A, Craddock N. Neurodevelopmental hypothesis of schizophrenia. Br J Psychiatry. 2011;198(3):173-5.

9. Khandaker GM, Cousins L, Deakin J, Lennox BR, Yolken R, Jones PB. Inflammation and immunity in schizophrenia: implications for pathophysiology and treatment. Lancet Psychiatry. 2015;2(3):258-70.

10. Khandaker GM, Zimbron J, Dalman C, Lewis G, Jones PB. Childhood infection and adult schizophrenia: a meta-analysis of population-based studies. Schizophrenia research. 2012;139(1-3):161-8.

11. Volk DW, Chitrapu A, Edelson JR, Roman KM, Moroco AE, Lewis DA. Molecular mechanisms and timing of cortical immune activation in schizophrenia. The American journal of psychiatry. 2015;172(11):1112-21.

12. Jeffries CD, Perkins DO, Fournier M, Do KQ, Cuenod M, Khadimallah I, et al. Networks of blood proteins in the neuroimmunology of schizophrenia. Translational psychiatry. 2018;8(1):112.

13. Keshavan M, Lizano P, Prasad K. The synaptic pruning hypothesis of schizophrenia: promises and challenges. World Psychiatry. 2020;19(1):110-1.

14. Pollak TA, Drndarski S, Stone JM, David AS, McGuire P, Abbott NJ. The blood-brain barrier in psychosis. Lancet Psychiatry. 2018;5(1):79-92.

15. Zheng P, Zeng B, Liu M, Chen J, Pan J, Han Y, et al. The gut microbiome from patients with schizophrenia modulates the glutamate-glutamine-GABA cycle and schizophrenia-relevant behaviors in mice. Sci Adv. 2019;5(2):eaau8317.

16. Cannon M, Jones PB, Murray RM. Obstetric complications and schizophrenia: historical and meta-analytic review. The American journal of psychiatry. 2002;159(7):1080-92.

17. Matheson SL, Shepherd AM, Pinchbeck RM, Laurens KR, Carr VJ. Childhood adversity in schizophrenia: a systematic meta-analysis. Psychol Med. 2013;43(2):225-38.

18. Khokhar JY, Dwiel LL, Henricks AM, Doucette WT, Green AI. The link between schizophrenia and substance use disorder: A unifying hypothesis. Schizophrenia research. 2018;194:78-85.

19. Vancampfort D, Firth J, Schuch FB, Rosenbaum S, Mugisha J, Hallgren M, et al. Sedentary behavior and physical activity levels in people with schizophrenia, bipolar disorder and major depressive disorder: a global systematic review and meta-analysis. World Psychiatry. 2017;16(3):308-15.

20. van Os J, Kenis G, Rutten BP. The environment and schizophrenia. Nature. 2010;468(7321):203-12.

21. Heinz A, Deserno L, Reininghaus U. Urbanicity, social adversity and psychosis. World Psychiatry. 2013;12(3):187-97.
